# Seven-year follow-up atherosclerotic plaque progression in patients with antiphospholipid syndrome versus diabetes mellitus and healthy controls

**DOI:** 10.1093/rheumatology/keae097

**Published:** 2024-02-06

**Authors:** Gerasimos Evangelatos, Nikolaos Tentolouris, Petros P Sfikakis, Maria G Tektonidou

**Affiliations:** Rheumatology Unit, First Department of Propaedeutic Internal Medicine, Joint Academic Rheumatology Program, Medical School, National and Kapodistrian University of Athens, Athens, Greece; Diabetes Centre, First Department of Propaedeutic Internal Medicine, Medical School, National and Kapodistrian University of Athens, Greece, Athens; Rheumatology Unit, First Department of Propaedeutic Internal Medicine, Joint Academic Rheumatology Program, Medical School, National and Kapodistrian University of Athens, Athens, Greece; Rheumatology Unit, First Department of Propaedeutic Internal Medicine, Joint Academic Rheumatology Program, Medical School, National and Kapodistrian University of Athens, Athens, Greece

**Keywords:** antiphospholipid syndrome, atherosclerotic plaques, carotid and femoral artery ultrasonography, cardiovascular disease, cardiovascular risk, systemic lupus erythematosus-associated APS

## Abstract

**Objectives:**

Patients with antiphospholipid syndrome (APS) carry a substantial burden of cardiovascular disease and subclinical atherosclerosis. We aimed to assess a 7-year follow-up atherosclerotic plaque progression in APS patients versus diabetes mellitus (DM) and healthy controls (HC).

**Methods:**

Eighty-six patients with thrombotic APS, 86 with DM and 86 HC (all age- and sex-matched) who underwent a baseline ultrasound of carotid and femoral arteries were invited for a 7-year follow-up ultrasonography examination. We compared atherosclerosis progression among the three groups and examined determinants of plaque progression in APS patients.

**Results:**

Sixty-four APS patients (75% females, 43.8% with primary APS), 58 patients with DM and 66 HC were included in the 7-year ultrasound re-evaluation. New plaque was detected in 51.6%, 36.2% and 25.8% of APS, DM and HC subjects, respectively. After adjusting for traditional cardiovascular risk factors (CVRFs) and baseline plaque presence, APS patients showed a 3-fold (OR = 3.07, *P* = 0.007) higher risk for atherosclerosis progression versus HC and 2-fold (OR = 2.25, *P* = 0.047) higher risk than DM patients. In multivariate analysis in the APS group, plaque progression was independently associated with systemic lupus erythematosus (SLE) co-existence (OR = 7.78, *P* = 0.005) and number of CVRFs (OR = 3.02, *P* = 0.002), after adjusting for disease-related parameters and CVRF-related medications. Sustained low-density lipoprotein target attainment reduced plaque progression risk (OR = 0.34, *P* = 0.021).

**Conclusion:**

Half of APS patients develop new atherosclerotic plaques over a 7-year follow-up, having a three-times higher risk versus HC. Concomitant SLE and number of traditional CVRFs are associated with plaque progression, supporting the need for thorough CVRF assessment and control.

Rheumatology key messages

- Half of antiphospholipid syndrome (APS) patients develop new atherosclerotic plaques over a 7-year period.
- APS patients exhibit a 3-fold risk for plaque progression compared to healthy individuals.
- Co-existent systemic lupus erythematosus and number of traditional cardiovascular risk factors predict atherosclerosis progression in APS.
- Sustained low-density lipoprotein control is inversely associated with plaque progression.

## Introduction

Antiphospholipid syndrome (APS) is a complex autoimmune disorder characterized by arterial and venous thrombotic complications, microvascular manifestations and pregnancy morbidity, in the presence of antiphospholipid antibodies (aPL). Cardiovascular disease (CVD) is a major cause of morbidity and mortality in these patients [1, 2]. Premature atherosclerosis is observed in autoimmune diseases, such as systemic lupus erythematosus (SLE) and APS [3, 4], where disease-related factors and traditional CVD risk factors (CVRFs) synergistically contribute to its development [3, 5, 6].

A growing body of evidence suggests a potential link between aPL and accelerated atherosclerosis [1, 4, 7]. APS thrombo-inflammation and atherogenesis share common intrinsic mechanisms involving aPL-mediated innate immunity dysregulation, oxidative stress and endothelial dysfunction, platelet activation and accumulation, thrombin generation, and in parallel, macrophage differentiation to foam cells [1, 7, 8]. We have previously shown an increased prevalence of subclinical atherosclerosis [9] and enhanced 3-years atherosclerosis progression in APS patients versus healthy controls (HC), in a degree comparable to diabetes mellitus (DM) [5].

Herein, we aimed to examine for the first time a 7-year progression of subclinical atherosclerosis in APS in comparison with HC but also with DM, a CVD risk equivalent disorder [10]. We additionally evaluated determinants of atherosclerosis progression among patients with APS.

## Methods

### Study design and population

All patients with thrombotic APS who were followed up in our Rheumatology Unit were assessed at baseline for eligibility to participate in this prospective vascular ultrasound study. APS patients fulfilled the revised Sapporo classification criteria for definite APS [11], while patients with SLE-APS also met the 2012 SLE classification criteria [12]. Individuals with active infection, active malignancy, history of atherosclerotic CVD event, concomitant DM, chronic kidney disease (CKD) of stage V or isolated obstetric APS were excluded from the study.

Carotid and femoral artery ultrasound was performed at baseline in 86 APS patients [43 with primary APS (PAPS) and 43 with SLE-APS], 86 age- and sex-matched DM patients, and 86 age- and sex-matched HC recruited by our Cardiovascular Research Laboratory using community-based methods. Three years after the baseline visit, a second vascular examination was conducted, and the results of this examination have been previously published [5]. All participants were invited for a third ultrasound evaluation 7 years after the baseline visit in order to assess subclinical atherosclerosis progression.

The study has been approved by the Institutional Review Board of our hospital (Laiko General Hospital Scientific Council No. 1041) and was conducted according to the principles of the Declaration of Helsinki. All participants provided written informed consent.

### Recorded parameters and ultrasound examination

Patient demographics and the following clinical data were recorded for all participants at baseline and follow-up visits: disease duration, family history of premature coronary artery disease (CAD), systolic and diastolic blood pressure (SBP and DBP, respectively), arterial hypertension (brachial SBP ≥140 mm or DBP ≥90 mmHg or antihypertensive treatment), dyslipidaemia (defined as low-density lipoprotein [LDL] over 160 mg/dl or lipid-lowering therapy), smoking status, exercise level (min per week), body mass index (BMI) and medications (statins, antihypertensives and antiplatelets). Measured laboratory data were serum creatinine, total cholesterol, high-density lipoprotein (HDL), LDL and triglycerides.

Disease-related parameters included the APS type (PAPS or SLE-APS), type of thrombotic events (arterial, venous), recurrence of thrombotic events, history of obstetric APS, aPL (lupus anticoagulant [LA], IgG and IgM isotypes of anti-cardiolipin [aCL] and anti-beta2-glycoprotein I antibodies [anti-β2GPI]), single, double or triple aPL positivity, high IgG or IgM aPL titers (>4-fold of the upper normal level in either aCL or anti-β2GPI), adjusted Global APS Score (aGAPSS), aGAPSS-CVD [13], and medications (anticoagulants, hydroxychloroquine [HCQ], corticosteroids, immunosuppressives). The Systemic Lupus Erythematosus Disease Activity Index 2000 (SLEDAI-2K) and the SLICC/ACR Damage Index (SDI) were calculated for SLE-APS patients for the assessment of disease activity and disease damage, respectively.

We also examined the attainment of therapeutic targets for blood pressure and dyslipidaemia, as addressed by the 2022 EULAR recommendations for CVD risk management [14] and the 2016 European Society of Cardiology (ESC) guidelines [15]. The BP and LDL target attainment was quantified using the number of visits in which the therapeutic target for each of the above CVRFs was achieved: none, 1, 2, 3 (considering the baseline, 3-year and 7-year ultrasound examination visits). For patients with PAPS, blood pressure target was defined as SBP <140 mmHg and DBP <90 mmHg, while for patients with concomitant SLE as SBP <130 mmHg and DBP <80 mmHg, according to EULAR recommendations for CVD management in SLE [14]. According to the ESC guidelines [15], the LDL target was defined according to CVD risk category (low, moderate, high or very high CVD risk) as it was classified by the Systemic Coronary Risk Evaluation (SCORE) prediction score. Participants with significantly elevated total cholesterol (>310 mg/dl) or blood pressure (SBP ≥180 or DBP ≥110 mmHg) were reclassified in high-risk category, while those with atherosclerotic plaque presence on carotid ultrasound were reclassified as the very high-risk [15].

For ultrasound examinations, a high-resolution device (Vivid 7 Pro, GE Healthcare) and a 14-MHz linear transducer was used. The same operator performed both baseline and follow-up vascular evaluations and was blinded to participants’ clinical data. Vascular measurements were performed in the near and far vessel walls in common carotid arteries, carotid bulbs, internal carotid arteries and common femoral arteries, as previously presented in detail [9]. The detection of a new atherosclerotic plaque either at carotid or femoral arteries at the follow-up examination, not identified at the baseline ultrasound test, was defined as atherosclerotic plaque progression.

### Statistical analysis

Categorical variables are presented as frequencies and percentages. Continuous variables are presented as either mean (±standard deviation, SD) or median (interquartile range, IQR), depending on their distribution. Normality of sample distribution was assessed using Kolmogorov–Smirnov test. Comparisons of categorical variables were performed by the Chi-squared test. For continuous variables, Student’s *t* test and ANOVA were applied for normally distributed variables, otherwise Mann–Whitney and Kruskal–Wallis tests were performed.

Multivariate logistic regression analysis was performed to compare plaque progression among the three groups (APS, DM, HC), adjusting for baseline presence of subclinical atherosclerosis and traditional CVRFs. To assess for possible predictors of plaque progression in APS patients, we performed multiple logistic regression analysis including variables found statistically significant in univariate analysis (Supplementary Table S1, available at *Rheumatology* online) or based on relevant evidence from the literature, such as arterial thrombosis, triple aPL positivity, anti-β2GPI positivity and high aPL titers [1, 9, 16]. To reduce the number of included variables in all models we utilized the variable ‘number of traditional CVRFs’, which incorporates high-risk age and sex groups (male over 55 years, female over 65 years), family history of premature CAD, arterial hypertension, dyslipidaemia, smoking and obesity. *P*-values <0.05 were considered as statistically significant. STATA, version 13.0 (College Station, TX, USA), was used for statistical analysis.

## Results

### Characteristics of study groups

From the total number of individuals examined by carotid/femoral ultrasound at baseline (all Caucasians), 64 APS patients, 58 DM patients and 66 HC were re-evaluated after 7 years (flowchart; Supplementary Fig. S1, available at *Rheumatology* online). The mean time between the baseline and the follow-up evaluation was 6.9 ± 1.1 years for APS patients, 6.2 ± 0.4 years for DM patients and 6.9 ± 0.6 for healthy subjects. From the APS population at baseline (*n* = 86, 50% with PAPS and 50% with SLE-APS), 28 (43.8%) of participants at 7-year follow-up had PAPS and 36 (56.2%) had SLE-APS. Among DM patients, 39.7% had type II DM, 77.6% were on insulin and the median HbA1c was 7.1 (6.8–8)%. Both APS and DM patients had a higher prevalence of traditional CVRFs compared with HC (Table 1).

**Table 1.** Characteristics of the three groups at baseline and at the end of follow-up

	APS (*n* = 64)	DM (*n* = 58)	HC (*n* = 66)	*P*-value
Baseline				
Age, years^a^	44.9 ± 10.7	47.4 ± 12.7	47.5 ± 12.6	**<0.001**
Sex (female), *n* (%)	48 (75.0)	42 (72.4)	49 (74.2)	0.946
Disease duration, years^a^	10.2 ± 8.6	15.4 ± 8.9	N/A	**0.001**
Family history of premature CAD, *n* (%)	3 (4.7)	11 (19.0)	5 (7.6)	**0.023**
Smoking, current, *n* (%)	20 (31.3)	26 (44.8)	18 (27.3)	0.102
Smoking, pack-years^a^	13.2 ± 18.2	11.6 ± 14.7	8.8 ± 13.4	0.256
Arterial hypertension, *n* (%)	17 (26.6)	28 (48.3)	24 (36.4)	**0.046**
SBP, mmHg^a^	123 ± 14	129 ± 19	126 ± 19	0.310
DBP, mmHg^a^	74 ± 9	76 ± 9	78 ± 12	**0.045**
Dyslipidaemia, *n* (%)	13 (20.3)	22 (37.9)	12 (18.2)	**0.025**
Total cholesterol, mg/dL^a^	186 ± 38	183 ± 34	206 ± 38	**0.004**
LDL, mg/dL^a^	110 ± 32	105 ± 33	126 ± 32	**0.001**
HDL, mg/dL^a^	54 ± 17	57 ± 18	59 ± 15	0.127
Triglycerides, mg/dL^b^	93 (71–142)	80 (55–150)	82 (65–129)	0.424
CKD (Stage III-IV), *n* (%)	3 (4.7)	6 (10.3)	1 (1.5)	0.088
BMI, kg/m^2^^a^	27.8 ± 5.5	27.8 ± 6.6	26.9 ± 5.1	0.636
Exercise level, min/week^b^	0 (0–180)	130 (0–315)	120 (0–225)	0.115
Anti-hypertensives, *n* (%)	18 (28.1)	25 (43.1)	16 (24.2)	0.061
Statins, *n* (%)	8 (12.5)	20 (34.5)	4 (6.1)	**<0.001**
Anti-platelets, *n* (%)	24 (37.5)	3 (5.2)	0 (0)	**<0.001**
Number of traditional CVRFs^b^	2 (1–2)	2 (1–3)	1 (0–2)	**0.016**
End of follow-up				
Follow-up, years^a^	6.9 ± 1.1	6.2 ± 0.4	6.9 ± 0.6	**<0.001**
Smoking, current, *n* (%)	18 (28.1)	17 (29.3)	13 (19.7)	0.398
Smoking, pack-years^a^	14.7 ± 20.3	13.3 ± 16.7	10.0 ± 14.5	0.295
Arterial hypertension, *n* (%)	23 (35.9)	33 (56.9)	31 (47.0)	**0.027**
SBP, mmHg^a^	125 ± 16	128 ± 16	126 ± 18	0.666
DBP, mmHg^a^	74 ± 8	73 ± 10	77 ± 11	0.067
Dyslipidaemia, *n* (%)	23 (35.9)	32 (55.2)	16 (25.4)	**0.003**
Total cholesterol, mg/dL^a^	180 ± 34	167 ± 29	202 ± 34	**<0.001**
LDL, mg/dL^a^	100 ± 29	90 ± 26	121 ± 30	**<0.001**
HDL, mg/dL^a^	59 ± 16	57 ± 17	60 ± 12	0.628
Triglycerides, mg/dL^b^	97 (74–130)	75 (59–123)	97 (71–140)	0.357
CKD (Stage III-IV), *n* (%)	4 (6.3)	10 (17.2)	2 (3.0)	**0.014**
BMI, kg/m^2^^a^	29.2 ± 5.9	28.2 ± 6.4	28.3 ± 5.7	0.484
Exercise level, min/week^b^	120 (0–300)	75 (0–180)	0 (0–210)	0.592
Anti-hypertensives, *n* (%)	25 (41.0)	30 (51.7)	26 (39.4)	0.279
Statins, *n* (%)	20 (31.3)	32 (55.2)	10 (15.2)	**<0.001**
Anti-platelets, *n* (%)	29 (45.3)	17 (29.3)	3 (4.5)	**<0.001**
Plaque presence at baseline, yes/no, *n* (%)	39 (60.9)	28 (48.3)	24 (36.4)	**0.020**
Plaque progession during follow-up, yes/no, *n* (%)	33 (51.6)	21 (36.2)	17 (25.8)	**0.010**

*P*-values refer to comparison between the three groups. Values in bold are statistically significant.

a Mean (SD).

b Median (IQR).

APS: antiphospholipid syndrome; BMI: body mass index; CAD: coronary artery disease; CKD: chronic kidney disease; CVRFs: cardiovascular risk factors; DBP: diastolic blood pressure; DM: diabetes mellitus; HC: healthy controls; HDL: high-density lipoprotein; LDL: low-density lipoprotein; SBP: systolic blood pressure.

Characteristics of patients with PAPS and SLE-APS are shown in Supplementary Table S2, available at *Rheumatology* online. SLE-APS patients had a median SLEDAI-2K of 2 (0–4) and a median SDI of 1 (0–2) at baseline, while at the end of follow-up the median SLEDAI-2K was 1 (0–2) and median SDI remained 1 (0–2). Patients with PAPS had a higher number of traditional CVRFs and a less frequent use of hydroxychloroquine compared with SLE-APS. The mean cumulative prednisone dose over the 7-year follow-up was 3.50 ± 5.86 g. Regarding CVRF control in APS, the blood pressure target was achieved in a higher degree than the LDL target (Supplementary Table S3, available at *Rheumatology* online).

### Progression of atherosclerosis

At the follow-up visit, new atherosclerotic plaques were observed in 33 (51.6%) of APS patients, 21 (36.2%) of DM patients and 17 (25.8%) of HC (Table 1). No plaque regression was observed. After adjusting for the number of traditional CVRFs and plaque presence at baseline evaluation, APS patients exhibited a 3-fold increased risk for plaque progression compared with HC (OR = 3.07, 95% CI 1.37–6.90, *P* = 0.007). Interestingly, APS patients had also higher risk than DM patients for new plaque formation during the 7-year follow-up (OR = 2.25, 95% CI 1.01–5.03, *P* = 0.047).

In the APS group, 39.1% of patients developed new plaques in carotid and 23.4% in femoral arteries. Atherosclerotic plaque progression occurred in 11 (39.3%) of PAPS and 22 (61.1%) of SLE-APS patients. In multivariate analysis, SLE-APS (OR = 7.78, 95% CI 1.83–33.05, *P* = 0.005) and number of traditional CVRF (OR = 3.02, 95% CI 1.50–6.06, *P* = 0.002), emerged as independent prognostic factors for new atherosclerotic plaque development during the 7-year follow-up (Table 2, Model 1). Similar results derived from two additional analyses adding anti-β2GPI positivity and high aPL positivity (Table 2, Model 2) or antihypertensives and statins use in the models (Table 2, Model 3). Importantly, LDL target attainment in more visits was independently associated with reduced risk of atherosclerosis progression (OR = 0.34, 95% CI 0.14–0.85, *P* = 0.021) (Table 2, Model 4). No association emerged between the duration of HCQ or the cumulative dose of corticosteroids and new plaque formation (Supplementary Table S1, available at *Rheumatology* online).

**Table 2.** Multivariate analysis for plaque progression within the group of APS patients.

	OR	95% CI	*P*-value
*Model 1*			
SLE-APS *vs* PAPS	7.78	1.83, 33.05	**0.005**
Arterial thrombotic events	2.64	0.78, 8.97	0.120
Triple aPL positivity	0.73	0.23, 2.39	0.608
CVRFs number	3.02	1.50, 6.06	**0.002**
*Model 2*			
SLE-APS *vs* PAPS	7.22	1.67, 31.22	**0.008**
Arterial thrombotic events	2.64	0.78, 8.94	0.119
Triple aPL positivity	0.59	0.10, 3.66	0.573
anti-β2GPI positivity	1.93	0.22, 17.08	0.555
High aPL positivity	0.58	0.12, 2.86	0.499
CVRFs number	3.14	1.50, 6.58	**0.002**
*Model 3*			
SLE-APS *vs* PAPS	10.03	2.08, 48.45	**0.004**
Arterial thrombotic events	3.99	0.94, 16.99	0.062
Triple aPL positivity	0.64	0.17, 2.37	0.503
Antihypertensives use	0.40	0.09, 1.71	0.216
Statins use	0.52	0.04, 6.24	0.603
CVRFs number	3.56	1.60, 7.92	**0.002**
*Model 4*			
SLE-APS *vs* PAPS	7.29	1.05, 50.41	**0.044**
Arterial thrombotic events	5.63	0.93, 34.05	0.060
Triple aPL positivity	2.57	0.33, 19.78	0.365
CVRFs number	3.16	1.04, 9.61	**0.043**
Number of visits with LDL target attainment (0, 1, 2, 3)^a^	0.34	0.14, 0.85	**0.021**
Number of visits with BP target attainment (0, 1, 2, 3)^b^	0.74	0.26, 2.11	0.570

Values in bold are statistically significant.

anti-β2GPI: anti-beta2-glycoprotein I antibodies; aPL: anti-phospholipid antibodies; APS: antiphospholipid syndrome; BP: blood pressure; CI: confidence interval; CVRFs: cardiovascular risk factors; LDL: low-density lipoprotein; OR: odds ratio; PAPS: primary APS; SLE: systemic lupus erythematosus.

a Number of visits in which the therapeutic target for LDL was achieved: 0 = none, 1 = 1 visit, 2 = 2 visits, 3 = 3 visits considering the baseline, 3-year follow-up and 7-year follow-up visits.

b Number of visits in which the therapeutic target for BP was achieved: 0 = none, 1 = 1 visit, 2 = 2 visits, 3 = 3 visits considering the baseline, 3-year follow-up and 7-year follow-up visits.

## Discussion

We assessed for the first time to our knowledge, the 7-year progression of atherosclerotic plaques in patients with APS compared with DM patients and healthy subjects. Our results revealed a 3-fold increased risk for plaque progression in APS patients versus HC, after adjusting for traditional CVRFs and baseline plaque presence. Interestingly, this risk was approximately double than that observed in individuals with DM, a prototype cardiovascular risk disorder.

Patients with APS have an increased CVD risk compared with the general population, while myocardial infarction and cerebrovascular events are recognized as leading causes of mortality [2]. A recent meta-analysis showed a four times higher risk of subclinical atherosclerosis in APS *vs* HC [4]. In the current study, subclinical atherosclerosis was prevalent in 61% of APS patients at the end of follow-up compared with 36.4% of HC, while new plaque formation (compared with the baseline examination) was detected in half (51.6%) of our APS population versus 25.8% of HC. After adjustment for potential confounders, APS patients were found to have three-times higher risk for new plaque formation within 7 years compared with HC. In a previous study from our group that examined the 3-year follow-up atherosclerosis progression, a similar 3.3-fold higher risk was demonstrated in APS patients versus HC [5]. Collectively, atherosclerotic process seems to be constantly accelerated in APS patients.

Atherosclerosis progression was more prevalent in SLE-APS than PAPS patients, in line with the results of our previous 3-year follow-up study [5]. Recent evidence has shown that SLE patients carry a 4-fold higher risk for atherosclerosis progression compared with HC over a 7-year period [3]. APLs exert a pro-atherogenic effect. Circulating aPLs induce an inflammatory milieu contributing to endothelial dysfunction and vascular remodeling promoting both thrombotic and atherosclerotic mechanisms [1, 7]. Similar mechanisms are involved also in SLE-related atherogenesis [17], in addition to the impact of several SLE-specific factors such as lupus nephritis and its characteristics (e.g. hypertension and/or nephrotic syndrome) or glucocorticoid exposure, contributing to the additive atherosclerotic burden of SLE-APS. Interestingly, we didn’t find any association between HCQ or cumulative corticosteroid dose and plaque progression. Use of antimalarials in SLE has been linked with reduction of CVD risk and is recommended for all SLE patients [14]. In a recent systematic review and meta-analysis, although antimalarials use was associated with a lower prevalence of several CVRFs (e.g. hypertension and diabetes mellitus) in SLE, no association was found with vascular ultrasonography markers such as carotid intima-media thickness (IMT) progression, carotid plaque and coronary artery calcification presence [18]. Corticosteroids is known to be proatherogenic [1] but the median cumulative dose in our APS population during the follow-up was low, reflecting the low SLE activity during the follow-up as expressed by the 3-year [5] and 7-year median SLEDAI-2K levels (2 [0–4] and 1 [0–2], respectively).

In the current study, the number of CVRFs at baseline was identified as an independent predictor of new atherosclerotic plaque development. Evidence from national registries and population-based data showed a high prevalence of traditional CVRFs in APS patients [19, 20]. However, poor CVRF target attainment was demonstrated in clinical practice [21]. This could be mainly attributed to the lower awareness of the treating physicians, although this has been gradually improved over the past years [21]. In contrast to rheumatoid arthritis and SLE where an increased CVD risk is well-established, the burden of atherosclerotic CVD in APS patients is less well recognized. Additionally, the excessive, for their young age, CVD risk is underestimated by the classical clinical prediction models [22]. We showed that sustained LDL target attainment significantly reduces atherosclerosis progression, highlighting the importance of early and rigorous CVRF assessment and management in APS patients, as urged by the latest EULAR recommendations for CVD risk management in rheumatic and musculoskeletal diseases, including SLE and APS [14]. Given that atherosclerotic plaque presence has high prognostic value for future CVD events and that clinical prediction models underestimate CVD risk in APS, vascular ultrasound may recognize high-risk patients who need a prompt intervention [22].

Our study has certain strengths: (i) this is the first study with such a long vascular ultrasound follow-up examination of a relatively adequate sample of APS patients, considering the rarity of syndrome and the lost to follow-up risk after long-time periods; (ii) the comparison with 1:1 age- and sex-matched HC, but also patients with DM, a prototype disorder of high CVD risk; (iii) all baseline and follow-up ultrasound examinations were performed by the same operator, reducing systematic bias. However, the single-centre cohort of predominantly Caucasians may limit the generalizability of the findings.

In conclusion, this study highlights the substantially increased long-term risk of atherosclerosis progression in APS patients compared with both DM patients and HC, especially in the SLE-APS group. The identification of traditional CVRF number as independent predictor of new plaque development underscores the importance of regular CVRF screening and prompt management in this high-risk population.

## Supplementary material

Supplementary material is available at *Rheumatology* online.

## Supplementary Material

keae097_Supplementary_Data

## Data Availability

The data that support the findings of this study are available from the corresponding author upon reasonable request.
