# Supplementary material for: Seven-year follow-up atherosclerotic plaque progression in patients with antiphospholipid syndrome versus diabetes mellitus and healthy controls
Source: Rheumatology (Oxford). 2024 Feb 6;64(2):836–41. doi: 10.1093/rheumatology/keae097 (PMC11781572; doi:10.1093/rheumatology/keae097)

**Supplementary Table S1**. Univariate analysis within the APS group, using plaque progression as dependent variable

|  | **OR** | **95% CI** | **p-value** |
| --- | --- | --- | --- |
| Age | **1.13** | **1.05, 1.22** | **0.001** |
| Sex (female vs male) | 0.38 | 0.12, 1.28 | 0.119 |
| Follow-up time | 1.16 | 0.74, 1.84 | 0.518 |
| Disease Duration (baseline) | 1.01 | 0.96, 1.08 | 0.818 |
| Family history of premature CAD | 0.45 | 0.04, 5.26 | 0.527 |
| Smoking, pack-years (baseline) | 1.01 | 0.98, 1.04 | 0.513 |
| Arterial hypertension (baseline) | 2.97 | 0.90, 9.78 | 0.073 |
| SBP (baseline) | **1.07** | **1.02, 1.12** | **0.009** |
| DBP (baseline) | 1.06 | 0.99, 1.13 | 0.061 |
| Dyslipidemia (baseline) | 2.53 | 0.69, 9.29 | 0.161 |
| Total cholesterol (baseline) | 1.01 | 0.99, 1.02 | 0.092 |
| LDL (baseline) | 1.02 | 0.99, 1.03 | 0.072 |
| HDL (baseline) | 0.99 | 0.96, 1.02 | 0.504 |
| Triglycerides (baseline) | 1.01 | 0.99, 1.01 | 0.241 |
| CKD (Stage III-IV) (baseline) | 1.94 | 0.17, 22.48 | 0.598 |
| BMI (baseline) | 1.05 | 0.96, 1.16 | 0.268 |
| Exercise (baseline) | 0.99 | 0.99, 1.00 | 0.705 |
| Traditional CVRF number (baseline) | **2.12** | **1.24, 3.64** | **0.006** |
| Anti-hypertensives use (baseline) | 1.25 | 0.42, 3.74 | 0.690 |
| Statins use (baseline) | 3.22 | 0.60, 17.4 | 0.173 |
| Antiplatelets (baseline) | 0.90 | 0.33, 2.49 | 0.846 |
| Number of visits with LDL target attainment (0, 1, 2, 3)* | **0.44** | **0.24, 0.79** | **0.006** |
| Number of visits with BP target attainment (0, 1, 2, 3)** | **0.49** | **0.27, 0.90** | **0.021** |
| Plaques presence (baseline) | 2.71 | 0.87, 8.40 | 0.085 |
|  | | | |
| APS type (SLE-APS vs PAPS) | 2.43 | 0.88, 6.68 | 0.086 |
| aCL IgG positivity (baseline) | 0.59 | 0.22, 1.61 | 0.305 |
| aCL IgM positivity (baseline) | 1.80 | 0.64, 5.09 | 0.267 |
| anti-β2GPI IgG positivity (baseline) | 0.79 | 0.28, 2.25 | 0.659 |
| anti-β2GPI IgM positivity (baseline) | 2.16 | 0.58, 8.07 | 0.252 |
| LA positivity (baseline) | 0.34 | 0.09, 1.23 | 0.101 |
| aPL positivity (single, double or triple) (baseline) | 0.71 | 0.37, 1.35 | 0.295 |
| High aPL titers (baseline) | 0.87 | 0.32, 2.33 | 0.777 |
| Recurrent Thromboses (baseline) | 0.79 | 0.29, 2.13 | 0.641 |
| Arterial thromboses (baseline) | 1.87 | 0.69, 5.07 | 0.221 |
| Obstetric APS (baseline) | 1.11 | 0.97, 1.27 | 0.133 |
| Corticosteroids, current (baseline) | 1.52 | 0.55, 4.14 | 0.418 |
| Cumulative prednisone dose (baseline) | 1.00 | 0.99, 1.00 | 0.536 |
| Cumulative prednisone dose (end of follow-up) | 1.00 | 0.99, 1.00 | 0.657 |
| HCQ, current (baseline) | 1.00 | 0.37, 2.68 | 0.994 |
| Cumulative duration of HCQ use (baseline) | 1.00 | 0.99, 1.01 | 0.104 |
| Cumulative duration of HCQ use (end of follow-up) | 1.00 | 0.99, 1.00 | 0.414 |
| Immunosuppressives, current (baseline) | 0.59 | 0.21, 1.70 | 0.332 |
| aGAPSS (baseline) | 1.02 | 0.90, 1.17 | 0.719 |
| aGAPSS-CVD (baseline) | 1.08 | 0.95, 1.23 | 0.258 |
| SLEDAI-2K (baseline) | 1.05 | 0.78, 1.40 | 0.767 |
| SDI (baseline) | 1.11 | 0.55, 2.28 | 0.766 |

Values in bold are statistically significant

APS: antiphospholipid syndrome, PAPS: Primary APS, SLE: Systemic Lupus Erythematosus, CAD: coronary artery disease, SBP: systolic blood pressure, DBP: diastolic blood pressure, LDL: low-density lipoprotein, HDL: high-density lipoprotein, CKD: chronic kidney disease, BMI: body mass index, CVRFs: cardiovascular risk factors, BP: blood pressure, aCL: anti-cardiolipin antibodies, anti-β2GPI: anti-beta2-glycoprotein I antibodies, LA: Lupus Anticoagulant, aPL: antiphospholipid, HCQ: hydroxychloroquine, aGAPSS: adjusted Global Anti-Phospholipid Syndrome Score, CVD: cardiovascular disease, SLEDAI-2K: Systemic Lupus Erythematosus Disease Activity Index 2000; SDI: Systemic Lupus International Collaborating Clinics-American College of Rheumatology Damage Index

***** Number of visits in which LDL was on therapeutic target: 0=none, 1=1 visit, 2=2 visits, 3=3 visits considering the baseline, 3-year follow-up and 7-year follow-up visits

** Number of visits in which BP was on therapeutic target: 0=none, 1=1 visit, 2=2 visits, 3=3 visits considering the baseline, 3-year follow-up and 7-year follow-up visits

|  | **PAPS** (n=28) | **SLE-APS** (n=36) | **p-value** |
| --- | --- | --- | --- |
| Age, years* | 45.3±12.0 | 44.6±9.7 | 0.715 |
| Sex (female), n (%) | 18 (64.3) | 30 (83.3) | 0.081 |
| Follow-up, years* | 6.8±1.1 | 6.9±1.1 | 0.968 |
| Disease Duration, years* | 8.5±7.7 | 11.5±9.1 | 0.244 |
| Family history of premature CAD, n (%) | 2 (7.1) | 1 (2.8) | 0.412 |
| Smoking, current, n (%) | 9 (32.1) | 11 (30.6) | 0.892 |
| Smoking, pack-years* | 15.2±18.5 | 11.6±18.1 | 0.567 |
| Arterial hypertension, n (%) | 7 (25) | 10 (27.8) | 0.803 |
| SBP, mmHg* | 123.6±11.6 | 123.0±16.2 | 0.867 |
| DBP, mmHg* | 73.0±7.4 | 74.6±9.5 | 0.631 |
| Dyslipidemia, n (%) | 6 (21.4) | 7 (19.4) | 0.845 |
| Total cholesterol, mg/dL* | 185.7±30.8 | 185.9±43.1 | 0.986 |
| LDL, mg/dL* | 111.0±32.9 | 109.4±32.3 | 0.856 |
| HDL, mg/dL* | 53.5±16.0 | 53.9±17.7 | 0.931 |
| Triglycerides, mg/dL⁑ | 94 (68-127) | 93 (75-145) | 0.511 |
| CKD (Stage III-IV), n (%) | 1 (3.6) | 2 (5.6) | 0.709 |
| BMI, kg/m^2^* | 29.2±4.4 | 26.8±6.0 | 0.079 |
| Exercise level, min/week⁑ | 0 (0-158) | 60 (0-195) | 0.453 |
| Number of CVRF | 2 (1-3) | 1 (1-2) | **0.029** |
| Anti-hypertensives, n (%) | 6 (21.4) | 12 (33.3) | 0.293 |
| Statins, n (%) | 6 (21.4) | 2 (5.6) | 0.057 |
| aCL IgG positivity, n (%) | 19 (67.9) | 29 (80.6) | 0.244 |
| aCL IgM positivity, n (%) | 14 (50.0) | 22 (61.1) | 0.374 |
| anti-β2GPI IgG positivity, n (%) | 14 (50.0) | 17 (47.2) | 0.825 |
| anti-β2GPI IgM positivity, n (%) | 11 (39.3) | 14 (38.9) | 0.974 |
| LA positivity, n (%) | 23 (82.1) | 27 (75.0) | 0.493 |
| aPL positivity  Single positivity, n (%)  Double positivity, n (%)  Triple positivity, n (%) | 6 (21.4)  9 (32.2)  13 (46.4) | 7 (19.4)  11 (30.6)  18 (50.0) | 0.774 |
| High aPL titers, n (%) | 18 (64.3) | 18 (50.0) | 0.253 |
| Recurrent Thromboses, n (%) | 15 (53.6) | 12 (33.3) | 0.104 |
| Arterial thromboses, n (%) | 19 (67.9) | 17 (47.2) | 0.099 |
| Venous thromboses, n (%) | 15 (53.6) | 22 (61.1) | 0.545 |
| Obstetric APS#, n (%) | 8 (44.4) | 9 (30.0) | 0.377 |
| Corticosteroids, current, n (%) | 3 (10.7) | 23 (63.9) | **<0.001** |
| Cumulative prednisone dose, g⁑ | 0 (0-1.46) | 7.56 (1.17 – 28.45) | **<0.001** |
| HCQ, current, n (%) | 8 (28.6) | 23 (63.9) | **0.005** |
| Cumulative duration of HCQ use, months* | 15.3±48.0 | 81.6±87.7 | **<0.001** |
| Immunosuppressives, current, n (%) | 5 (17.9) | 16 (44.4) | **0.025** |
| Anticoagulants, n (%) | 25 (89.3) | 27 (75.0) | 0.146 |
| Antiplatelets, n (%) | 8 (28.6) | 16 (44.4) | 0.193 |
| aGAPSS* | 9.5±3.7 | 9.0±3.9 | 0.507 |
| aGAPSS-CVD* | 11.0±3.8 | 10.4±4.1 | 0.534 |
| SLEDAI-2K⁑ | N/A | 2 (0-4) | N/A |
| SDI⁑ | N/A | 1 (0-2) | N/A |

**Supplementary Table S2**. Baseline characteristics of patients with PAPS and SLE-APS

Values in bold are statistically significant

*Mean (SD), ⁑Median (IQR)

# Calculated for the group of female patients (n=18 for PAPS, n=30 for SLE-APS)

APS: antiphospholipid syndrome, PAPS: Primary APS, SLE: Systemic Lupus Erythematosus, CAD: coronary artery disease, SBP: systolic blood pressure, DBP: diastolic blood pressure, LDL: low-density lipoprotein, HDL: high-density lipoprotein, CKD: chronic kidney disease, BMI: body mass index, CVRFs: cardiovascular risk factors, aCL: anti-cardiolipin antibodies, anti-β2GPI: anti-beta2-glycoprotein I antibodies, LA: Lupus Anticoagulant, aPL: antiphospholipid, HCQ: hydroxychloroquine, aGAPSS: adjusted Global Anti-Phospholipid Syndrome Score, CVD: cardiovascular disease, SLEDAI-2K: Systemic Lupus Erythematosus Disease Activity Index 2000; SDI: Systemic Lupus International Collaborating Clinics-American College of Rheumatology Damage Index

**Supplementary Table S3**. Target attainment for LDL and blood pressure in APS group

|  | LDL (n=45) | Blood pressure (n=64) |
| --- | --- | --- |
| 0 visit (0%) | 14 (31.3%) | 5 (7.8%) |
| 1 visit (33.3%) | 11 (24.4%) | 8 (12.5%) |
| 2 visits (66.7%) | 8 (17.8%) | 17 (26.6%) |
| 3 visits (100%) | 12 (26.7%) | 34 (53.1%) |

LDL: low-density lipoprotein

**Supplementary Figure S1**. Study flowchart


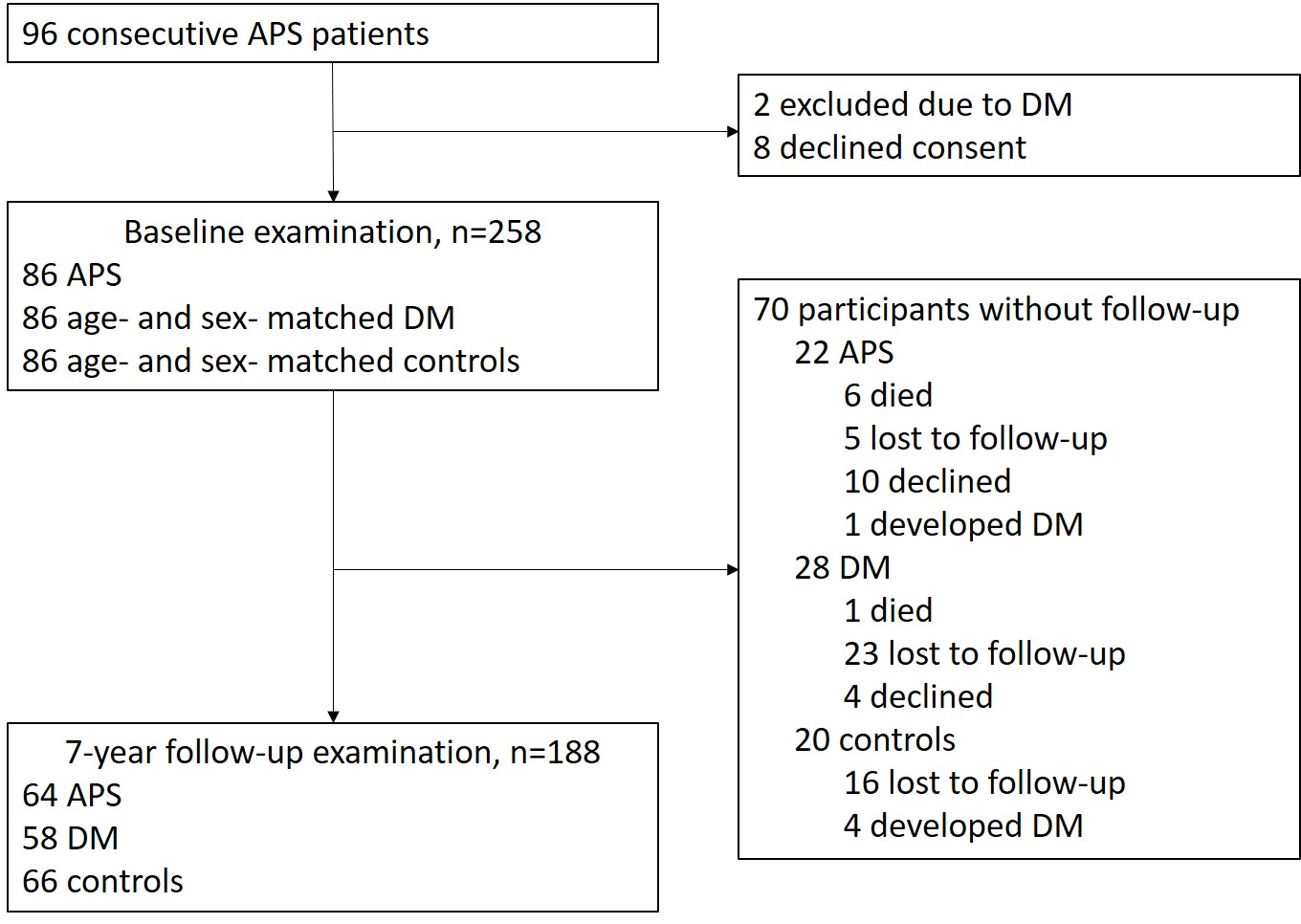

Supplement: keae097_Supplementary_Data [file keae097_supplementary_data.docx]
